# Kinin B1 receptor deficiency protects mice fed by cafeteria diet from abnormal glucose homeostasis

**DOI:** 10.1371/journal.pone.0267845

**Published:** 2022-05-26

**Authors:** Poliana E. Correia, Clarissa B. Gomes, Vinicius A. Bandeira, Thais Marten, Gabriella R. Natividade, Paula Merello, Erica Tozawa, Carlos T. S. Cerski, Alexandre Budu, Ronaldo Araújo, Bruno D. Arbo, Maria Flávia M. Ribeiro, Carlos C. Barros, Fernando Gerchman

**Affiliations:** 1 Graduation Program in Medical Science: Endocrinology, Department of Internal Medicine Faculty of Medicine, Universidade Federal do Rio Grande do Sul, Porto Alegre, Brazil; 2 Faculty of Nutrition, Federal University of Pelotas, Pelotas, Brazil; 3 Universidade Federal do Rio Grande do Sul, Porto Alegre, Brazil; 4 Pathology Division, Hospital de Clínicas de Porto Alegre, Universidade Federal do Rio Grande do Sul, Porto Alegre, Brazil; 5 Department of Biophysics, Universidade Federal de São Paulo, São Paulo, Brazil; 6 Departament of Physiology, Institute of Basic Health Sciences, Universidade Federal do Rio Grande do Sul, Porto Alegre, Brazil; 7 Endocrine and Metabolism Division, Hospital de Clínicas de Porto Alegre, Universidade Federal do Rio Grande do Sul, Porto Alegre, Brazil; Auburn University, UNITED STATES

## Abstract

The kallikrein–kinin system has been implicated in body weight and glucose homeostasis. Their major effectors act by binding to the kinin B2 and B1 receptors. It was assessed the role of the kinin B1 receptor in weight and glucose homeostasis in B1 receptor knockout mice (B1RKO) subjected to a cafeteria diet (CAF). Wild-type (WT) and B1RKO male mice (C57BL/6 background; 8 weeks old) were fed a standard diet (SD) or CAF for 14 weeks, *ad libitum*, and four groups were formed: WT-SD; B1RKO-SD; WT-CAF; B1RKO-CAF. Body weight and food intake were assessed weekly. It was performed glucose tolerance (GTT) and insulin tolerance tests (ITT), and HOMA-IR, HOMA-β and HOMA-β* 1/HOMA-IR were calculated. Islets from WT and B1RKO were isolated in order to measure the insulin secretion. Western blot was used to assess the hepatic AKT phosphorylation and qPCR to assess gene expression. CAF induced a higher body mass gain in B1RKO compared to WT mice. CAF diet increased epididymal fat depot mass, hepatic fat infiltration and hepatic AKT phosphorylation in both genotypes. However, B1RKO mice presented lower glycemic response during GTT when fed with CAF, and a lower glucose decrease in the ITT. This higher resistance was overcomed with higher insulin secretion when stimulated by high glucose, resulting in higher glucose uptake in the GTT when submitted to CAF, despite lower insulin sensitivity. Islets from B1RKO delivered 4 times more insulin in 3-month-old mice than islets from WT. The higher insulin disposition index and high insulin delivery of B1RKO can explain the decreased glucose excursion during GTT. In conclusion, CAF increased the β-cell function in B1RKO mice, compensated by the diet-induced insulin resistance and resulting in a healthier glycemic response despite the higher weight gain.

## Introduction

Obesity is caused by the disruption of energy homeostasis, leading to ectopic fat deposition, fatty liver disease, diabetes, and their complications [1, 2]. The activation of different pathways leading to inflammation is a link between body weight gain and its comorbidities [3]. In this context, the kallikrein–kinin system (KKS) has been identified as having a possible role in the development of obesity [4–7]. Kinins, bradykinin (BK) and lys-bradykinin (Lys-BK), plus their metabolites des-Arg^9^-bradykinin (DBK) and Lys-des-Arg^9^-bradykinin, activate two G protein–coupled receptors, the B1 and B2 kinin receptors [8]. The B2 receptor (B2R) is constitutively expressed in different tissues, and it is downregulated following ligand binding, leading to a rapid desensitization [8]. On the other hand, B1 receptor (B1R) is weakly expressed in physiological conditions in different tissues [8], but its expression is induced by inflammation and pro-inflammatory cytokines [9–11].

B1 receptor knockout mice (B1RKO) were resistant against weight gain and presented a significant decrease in the leptin content, improving the leptin sensitivity, and decreasing the hepatic lipid accumulation while subjected to a specific high-fat diet (HFD), when compared to controls [5, 12]. Nevertheless, there was no reduction of the inflammation markers in B1RKO under HFD, such as interleukins (ILs) -6 and -11 nor was there an increase in the anti-inflammatory interleukin IL-10 in those mice [12]. B1RKO also showed a lower insulin content in isolated islets, higher insulin sensitivity, and lower fasting plasma glucose 2h after feeding, when compared to wild-type (WT) controls [12–15]. In agreement with this, blockade of the B1 receptor by the specific inhibitor SSR240612 reverted hyperinsulinemia and hyperglycemia to baseline levels in insulin-resistant glucose-fed rats [7]. Those findings suggest changes in the insulin signaling, production and release.

The cafeteria diet (CAF) mimics a high palatability and energy-dense diet, usually associated to the development of obesity. In Western societies, CAF is composed by foods with high content of energy, simple carbohydrates, refined sugar, high saturated fats and trans fats. CAF also presents low protein, micronutrient, and fiber content [16]. Its composition, high palatability and high-energy contents disrupt normal appetite regulation, leading rodents to increase their calorie intake by 30 to 40% as compared to animals fed a high-lipid diet (HFD) [17], as well as inducing metabolic dysfunctions such as hyperglycemia, hyperinsulinemia, and high plasma no esterified fatty acids in comparison to HFD [17–19]. All those differences between such diets can challenge genetic variations in completely different ways.

Since B1RKO has never been exposed to CAF before, the purpose of the present study was to investigate the role of the B1 receptor deletion on body weight, glucose metabolism, and visceral lipid accumulation in mice exposed to a hyper-palatable cafeteria diet.

## Material and methods

Experimental diets and *in vivo* analysis were carried out at the Laboratory of Experimental Nutrition of Universidade Federal de Pelotas (UFPel). After euthanasia, biological materials were analyzed in the Molecular Biology laboratory of the Endocrinology Unit at Hospital de Clínicas of Porto Alegre (HCPA), the Universidade Federal do Rio Grande do Sul (UFRGS). All research and animal care procedures were conducted in agreement with international guidelines, and were approved by the institutional review boards of all facilities involved in the study (UFPel, 3913/2016; HCPA, 16–0397; UFRGS, 33191).

### Animals

Experiments were carried out with Bdkrb1^tm^/Bdkrb1^tm^ male mice (B1RKO) and their wild-type controls (WT), both with C57BL/6 background and 8 weeks old, obtained from the Federal University of São Paulo vivarium. Animals were kept in plastic cages on a ventilated shelf with controlled humidity (40–60%) and temperature (22 ± 2°C) at a 12/12h light-dark cycle. Before starting the experimental diets, all animals had *ad libitum* access to standard chow and tap water. For the experiment, B1RKO and control mice were randomized according to the maternal background in two groups fed with CAF or standard diet (SD) for 14 weeks, as follows: WT-SD (n = 7), B1RKO-SD (n = 8), WT-CAF (n = 7), and B1RKO-CAF (n = 10). Isoflurane anesthesia was used before the euthanasia, in order to avoid suffering when there was no surgery in the procedures, thus eliminating the need for any application of drugs for analgesia.

### Experimental diets

The standard diet consisted of rodent chow (Nuvilab CR-1, Nuvital, Curitiba, Brazil) with the following estimated composition: 69% carbohydrate, 26% protein, and 5% lipids (energy density: 3 kcal/g). CAF diet was an adaptation of the model described by Estadella et al. (2004) [16] and Macedo et al. [20]. It consisted of standard rodent chow, the same offered to the control group added to potato chips, bacon, cookies, condensed milk, and soda, with the following composition: 52–55% carbohydrate, 12–13% protein, and 33–34% lipids. Animals in the CAF group had at their disposal three drinking sources: no-gas soda, condensed milk diluted in water, and pure water, among which they could choose. Those food components were supplied daily in their natural form, and animals were free to select and consume them. Food and total liquid intake were daily assessed at 9:00am by measuring the difference in the food weighed in the previous day and the leftovers after 24 hours. The result was obtained by splitting such amount by the number of animals in each living box and expressed by the average animal intake. Animals were individually weighed once a week. The amount of food consumed was converted into energy by using data from the manufacturers and food tables.

### Assessment of insulin sensitivity, peripheral glucose uptake, and β-cell function

A glucose tolerance test (GTT) was performed after an 8-hour fast in the last week of the dietary intervention. Glycemia was measured in tail blood by the use of a glucometer (Accu-Chek Performa, ROCHE, Basel, Switzerland) before and after intraperitoneal injection of 1 g/kg body weight (BW) of 10% glucose solution: at -15, 0, 15, 30, 60 and 120 minutes after injection. Analyses were made by comparison of the area under the curve (AUC) of each group by the trapezoid method [21]. For the insulin tolerance test (ITT) performed two days after the GTT test, animals had a 2-hours fast. Glycemia was measured before and after intraperitoneal injection of 1 IU/kg BW of regular insulin, at -15, 0, 5, 20, and 30 minutes after injection. The insulin decrease was calculated by using the constant rate of glucose disappearance (K_ITT_) and presented as percentage of glucose decrease/minute. The K_ITT_ was calculated between 5 and 20 minutes after the insulin inoculation, in order to avoid the influence of the initial adrenaline discharge and the delayed release of hyperglycemic hormones after the reduction of the blood glucose caused by the insulin.

### Euthanasia

Animals were fasted overnight and euthanized by decapitation after deep anesthesia with isoflurane. Abdominal adipose tissue (epididymal and perirenal) and liver were manually dissected, weighted, flash-frozen in dry ice, and stored at -80°C. Liver fragments were also conserved in a 10% formalin solution for histological analysis.

### Biochemical analysis

After the euthanasia, the blood was collected in micro tubes containing EDTA, and the plasma was separated from the cells. The fractions were frozen and stored at -80°C. Plasma levels of insulin were measured by ELISA (catalog # EZRMI-13K, Millipore, Billerica, MA, USA), following instructions of the manufacturer. Plasma levels of glucose were measured at the HCPA Clinical Pathology Unit by using the enzymatic UV–hexokinase method (Cobas c702). To analyze the β-cell response to insulin sensitivity, it was calculated the disposition index (DI = β-cell function response adjusted to the level of insulin sensitivity [HOMA-β * 1/HOMA-IR]) [22–24].

### Histological analysis

Liver sections were stored in buffered formalin and stained with hematoxylin and eosin for histological analysis. In short: sections were fixed in formalin and wrapped in paraffin, the blocks were dehydrated in a graded series of ethanol and embedded in paraffin wax. Serial 3 μm thick sections were stained with hematoxylin and eosin. Sections were examined for NAFLD-specific lesions by two experienced pathologists, blinded to genotype and diet intervention. Steatosis, ballooning, and steatohepatitis were assessed to generate a semi quantitative NAFLD activity score, ranging from 0–8. Steatosis was graded as the percentage of hepatocytes that were steatotic: 1, <5%; 2, 5–33%; 3, 34–66%; and 3, >66%. Ballooning was scored as: 0, absent; 1, mild; 2, moderate; and 3, severe. Lobular inflammation was scored as: 0, no foci; 1, <2 foci per 200x field; 2, 2–4 foci per 200x field; and 3, >4 foci per 200x field. The final total score was used to classify the level of liver injury by using the NAFLD activity score (NAS) [25]; the higher the score, the more severe the liver disease. In the reference study, NAS scores of 0–2 occurred in cases largely considered not NASH diagnostic, scores of 3–4 were evenly divided among those considered not diagnostic, borderline, or positive for NASH, and scores of 5–8 occurred in cases largely considered NASH diagnostic [25].

### RNA isolation and gene expression

Liver fragments were homogenized in phenol-guanidine isothiocyanate (Trizol^®^ Reagent, Invitrogen, Carlsbad, CA, USA). RNA was extracted with chloroform and precipitated with isopropanol by centrifugation (12,000×*g*) at 4°C. The RNA pellet was washed twice with 75% ethanol and resuspended in 40–80 μL of diethyl pyrocarbonate-treated water. Concentration and quality of total RNA samples were assessed by using a NANODROP 2000 spectrophotometer (Thermo Fisher Scientific, Waltham, MA, USA). For the genic expression analysis, RNA was reverse-transcribed by using a SuperScript^®^ VILO^™^ cDNA Synthesis Kit (Life Technologies, Carlsbad, CA, USA), following the protocols provided by the manufacturer. Quantitative PCR was performed by monitoring the increasing fluorescence of Fast SYBR^®^ Green Master Mix (Life Technologies, Carlsbad, CA, USA). For the reaction, a 7500 Fast Real-Time PCR System Thermal Cycler (Life Technologies, Carlsbad, CA, USA) was used. Specific primer sequences for the genes encoding glucose-6-phosphatase, glucokinase, phosphoenolpyruvate, carboxykinase, fructose 1,6-bisphosphatase, and hepatocyte nuclear factor 4-alpha were designed and tested in the samples by analyzing the curve patterns, in order to set the amplification effectiveness of each primer pair. For the Kinin B2 receptor expression Quantitative PCR was performed with the TaqMan system (Applied Biosystems, Carlsbad, CA) and specific kit of oligonucleotides (B2R-Forward-5-GGT GCT GAG GAA CAA CGA GA-3, B2R-Reverse-5-CCC AAC ACA GCA CAA AGA GC-3, the control gene was the Glyceraldehyde-3-phosphate dehydrogenase (GadDH)—Forward-5-GCT GTG GGC AAG GTC ATC C-3 and Reverse-5-CTT CAC CAC CTT CTT GAT GTC-3) [26], and the thermic protocol was as follows: holding 95°C for 10 min, and a cycle of 95°C for 30 s, 60°C for 30 s, and 72°C for 30 s repeated 40 times. Gene expression was normalized against the GadDH gene expression and defined as relative values utilizing the threshold cycle method (CT; 2-ΔΔCt), following instructions from the manufacturer [27].

### Western blot

Liver samples were homogenized in lysis buffer (pH 7.4) containing protease inhibitors and detergents. The homogenates were centrifuged at 7,000×*g* for 10 min at 4°C to discard cell debris, and the supernatant fraction obtained was used for the Western blot assay. Protein levels were measured by the method of Bradford [28]. Electrophoresis and protein transfer were performed as described elsewhere [29]. The membranes were processed for immunodetection by using rabbit polyclonal antibodies for p-AKT and AKT (60 kDa) (1:500 dilution) (Santa Cruz Biotechnology, Santa Cruz, CA, USA). After washing with TTBS, the membranes were incubated for 2h at room temperature with goat anti-rabbit antibody (Millipore, Burlington, MA, USA) (1:10,000 dilution) and washed with TBS (20 mM Tris-HCl, 140 mM NaCl, pH = 7.4). Detection of protein bands was performed by chemiluminescence followed by exposure to autoradiography film. Densitometric analysis of the autoradiography was performed on ImageJ software (NIH, Bethesda, MD, USA). To minimize the inter-assay variation, samples from all experimental groups were processed in parallel. Protein expression values were calculated as arbitrary densitometric units [30].

### Insulin secretion in isolated islets

In a second set of trial, B1RKO and WT male mice aging between 3 and 6 months and fed SD were used to prepare the pancreas for the islet collection and *in vitro* islet culture. This previously method was described by BARROS et al [27]. After deep anesthesia with isoflurane, the pancreas and duodenum were exposed, and the common bile duct was cannulated. The exocrine pancreas was digested by retrograde 3 mL collagenase solution infusion at 0.2 U/mL (C9263-1G, Sigma, USA). Inflated pancreas was incubated for 11 minutes at 37°C. Next, the reaction was stopped by adding ice-cold balanced salt solution by Hank (4°C) and 4 sequential washes. The islets were manually selected among the cell debris by using a Pasteur pipette. Ten pancreatic islets were initially pre-incubated for 45 min at 37°C in Krebs-Ringer bicarbonate buffer with the following composition (in mmol/l): NaCl, 115 mM; KCl, 5 mM; CaCl2, 2.56 mM; MgCl2, 1 mM; NaHCO3, 24 mM, and glucose, 5.6 mM, supplemented with BSA (0.3% w:v) and balanced with a 95% O2:5% CO2, pH 7.4 mixture. The solution was then replaced, and the islets incubated for 90 min under the experimental conditions (2.8 and 22.4 mM of glucose) for 1 hour. Insulin concentration was measured by ELISA (EZRMI-13K, Rat/Mouse Insulin ELISA, Sigma-Aldrich, USA).

### Statistical analysis

Data are expressed as mean ± standard error of the mean (SEM). Statistical analyses were carried out by using one-way and two-way analysis of variance (ANOVA) or covariance (ANCOVA) followed by the Bonferroni post-test using log_10_ values of each group, whenever required in order to minimize the effects of nonparametric distribution and to be more conservative as to significant findings. Those statistical approaches are considered more adequate to understand differences of measurements between groups because it not only takes into account changes from baseline over time after an experiment in a group, but also differences of the variation of those measurements over time between groups and their genotype [31]. Weight gain was calculated by subtracting the initial weight of each animal from its final weight. The result was normalized by the initial weight and expressed as a percentage. The interaction between the diet intervention and genotypes over time was analyzed by using generalized estimating equations (GEE), a robust method for between-groups variance, including diet, genotype, time, and the group-by-time interaction as predictors. Statistical analyses of this data were calculated in PASW Statistics, Version 18 (SPSS Inc., Chicago, IL, USA), and plotted on GraphPad Prism 8.0 (GraphPad Software, La Jolla, CA, USA). The significance level was set at 5% (p<0.05).

## Results

To assess the role of the CAF diet, it was analyzed the daily calorie intake, macronutrients, sugary beverages and water along the experiment. Mice fed with CAF presented higher total and relative energy intake and higher consumption of lipids in comparison to those fed with SD for both genotypes, as expected (kcal/week = 109.17 ± 2.32 vs 209.21 ± 7.81, WT-SD vs WT-CAF; 126.48 ± 3.27 vs 195.49 ± 6.55, B1RKO-SD vs B1RKO-CAF; p <0.05). Upon comparing the genotypes, total and relative energy intake (kcal/week and kcal/g BW) were higher in B1RKO than in WT mice on SD, but not when fed CAF (p < 0.05) (Table 1). While lipid intake increased in both genotypes on CAF diet, the protein and water intake was reduced with this diet, as animals had other sugary drinks available. The intake of complex carbohydrate was also reduced in CAF, as the lipid percentage was higher and the total carbohydrate refined sugar intake was higher in CAF in the form of sugar-sweetened beverages (< 0.05). Water and protein intake were lower in B1RKO than in WT mice fed by CAF, whereas carbohydrate and lipid intake were similar between both genotypes (Table 1).

**Table 1:** Dietary composition of the experiment according to group interventions.

PARAMETER	WT-SD	B1RKO-SD	WT-CAF	B1RKO-CAF
Energy intake (kcal/week)	109.17 ± 2.32	126.48 ± 3.27*	209.21 ± 7.81^#^	195.49 ± 6.55 ^#^
Kcal/g body weight	3.6 ± 0.05	4.41 ± 0.10*	6.45 ± 0.19^#^	6.55 ± 0.24^#^
Carbohydrate, % of energy	69	69	52.12 ± 1.03^#^	54.29 ± 0.56^#^
Protein, % of energy	26	26	13.13 ± 0.21^#^	12.21 ± 0.23^#^*
Lipid, % of energy	5	5	34.18 ± 0.93^#^	33.12 ± 0.59^#^
Water intake (mL/week)	43.74 ± 1.54	45.53 ± 1.19	19.88 ± .92^#^	12.56 ± 0.66^#^*
Sugar-sweetened beverages	0	0	73.8 ± 5.33^#^	69.01 ± 4.26^#^

Data are expressed as mean ± SEM of the weekly values obtained from each box divided by the number of animals living in the box. In the CAF diet, foods were given *in natura*, and the macronutrient intake thus varied each week. P-values by one-way ANOVA followed by the Bonferroni post-hoc test. Statistical comparison between WT x B1RKO in the same diet (*) or SD x CAF in the same genotype.

^(#)^ indicates p < 0.05.

WT, wild type; B1RKO, B1R knockout mice; SD, standard diet; CAF, cafeteria diet, WT-SD (n = 7), B1RKO-SD (n = 8), WT-CAF (n = 7), and B1RKO-CAF (n = 10).

### Body weight according to genotype and diet

**Fig 1:**
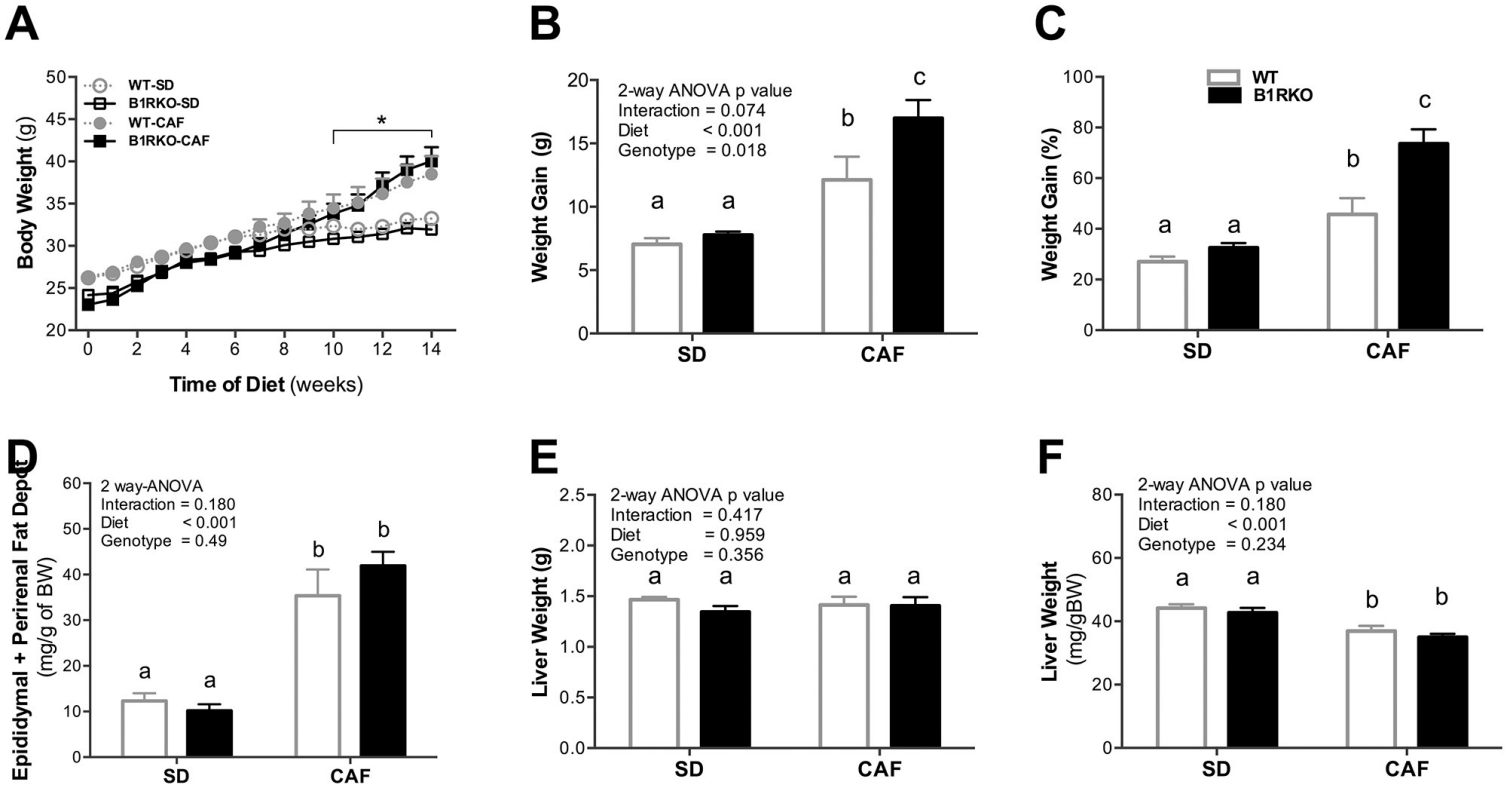
Changes in body weight, epididymal and perirenal fat, and liver weight according to diet and genotype. **A)** Weekly body weight over the 14-week diet protocol. **B and C**) Weight gain. **D)** Relative epididymal and perirenal fat pad weight is higher in CAF animals compared to SD animals. **E)** Absolute liver weight. **F)** Relative liver weight. P-value by two-way ANOVA followed by Bonferroni post-hoc test. Different letters indicate differences in post-hoc test. Data presented as mean ± SEM. WT, wild type; B1RKO, B1R knockout mice; SD, standard diet; CAF, cafeteria diet, WT-SD (n = 7), B1RKO-SD (n = 8), WT-CAF (n = 7), and B1RKO-CAF (n = 10).

It was compared the changes in body weight and weight gain according to the diet intervention and genotype. There were no differences in body weight according to genotype, except for different diets (Fig 1A). The weight gain increased in mice under CAF (Fig 1B and 1C). B1RKO had higher body weight than WT under CAF (Fig 1B and 1C). At the end of the diet intervention, mice under CAF presented higher epididymal and perirenal fat pad weight (Fig 1D). Such relationships were not affected by genotypes nor diet-genotype interactions (Fig 1D). Liver mass presented no differences between diets nor genotype (Fig 1E and 1F).

### Absence of kinin B1 receptor protects mice fed by CAF from a higher glucose excursion

**Fig 2:**
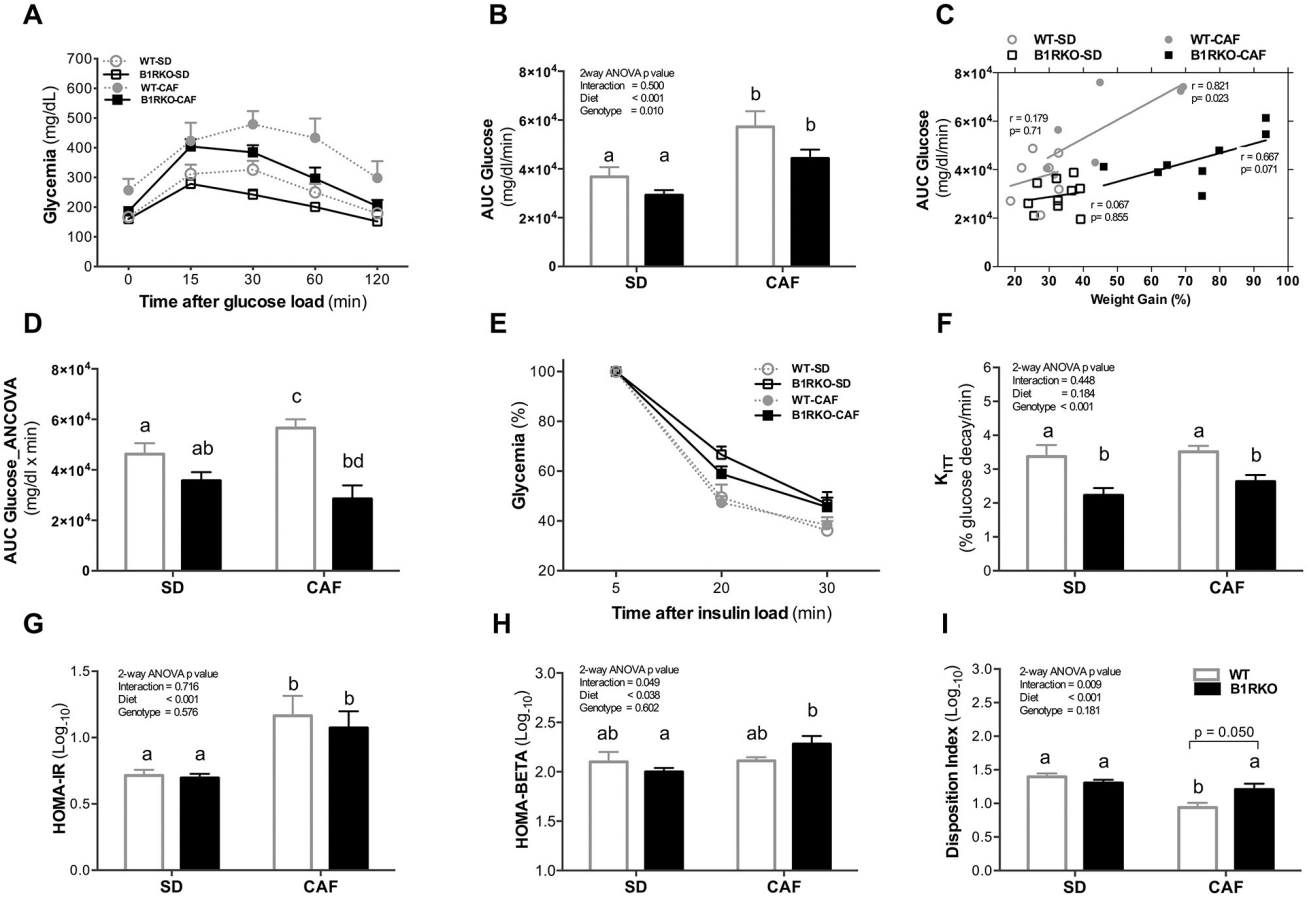
Glycemic response to dynamic tests and insulin sensitivity/ β-cell function according to diet and genotype. **A)** GTT plasma glucose curve. **B)** AUC of glucose (ANOVA). **C)** Correlation between weight gain (%) and AUC in both the SD and CAF groups tested by the Correlation of Spearman. **D)** The glucose AUC was significantly higher in WT-CAF compared to B1RKO-CAF and WT-SD. P-value tested by the ANCOVA. **E and F)** Absolute and relative values of the insulin tolerance test (ITT). **G to I)** HOMA indexes to assess insulin resistance and β-cell function. P-value tested by two-way ANOVA followed by the Bonferroni post-hoc test. Different letters indicate differences in the post-hoc test. Data are expressed as mean ± SEM. WT, wild type; B1RKO, B1R knockout mice; SD, standard diet; SD; CAF, cafeteria diet, WT-SD (n = 7), B1RKO-SD (n = 8), WT-CAF (n = 7), and B1RKO-CAF (n = 10).

In order to analyze glycemic responses according to the diet and genotype, mice were exposed to an intraperitoneal glucose load (Fig 2A). Basal glycemia was lower in B1RKO-CAF compared to WT-CAF (Fig 2A). CAF induced a higher glycemic response compared to SD and corrected by the body weight gain (area under the curve: CAF = 50768 ± 2874 glucose x minute vs. SD = 33004 ± 2737; p = 0.001 for diet in two-way ANOVA). This effect was lower in B1RKO than in WT mice, and no interaction was observed between genotype and diet (Fig 2B). As the glucose response was lower in B1RKO than in WT mice regardless the diet, it was analyzed how the weight changes influenced the response to glucose injection in relation to the genotype and diet. While glycemic responses were not related to weight gain in mice receiving SD, glycemic response was strongly related to the weight gain in WT mice, and moderately related to the weight gain in B1RKO mice receiving CAF (Fig 2C). Furthermore, upon analyzing how the higher weight gain affected glycemic response by genotype in mice fed by CAF, it was built a statistical model adjusting the differences in weight gain between groups. B1RKO-CAF mice were found to have a lower adjusted glycemic response than WT-CAF mice adjusting the weight (WT-SD 46.246 ± 4.302 vs. B1RKO-SD 35.699 ± 3.383; p = 0.03; and WT-CAF 56.564 ± 3.477 vs. B1RKO-CAF 28.521 ± 5.338; AUC ANCOVA, p = 0.0001) (Fig 2D).

### Mechanisms underlying the role of the kinin B1 receptor on glucose homeostasis in mice receiving a cafeteria diet

**Fig 3:**
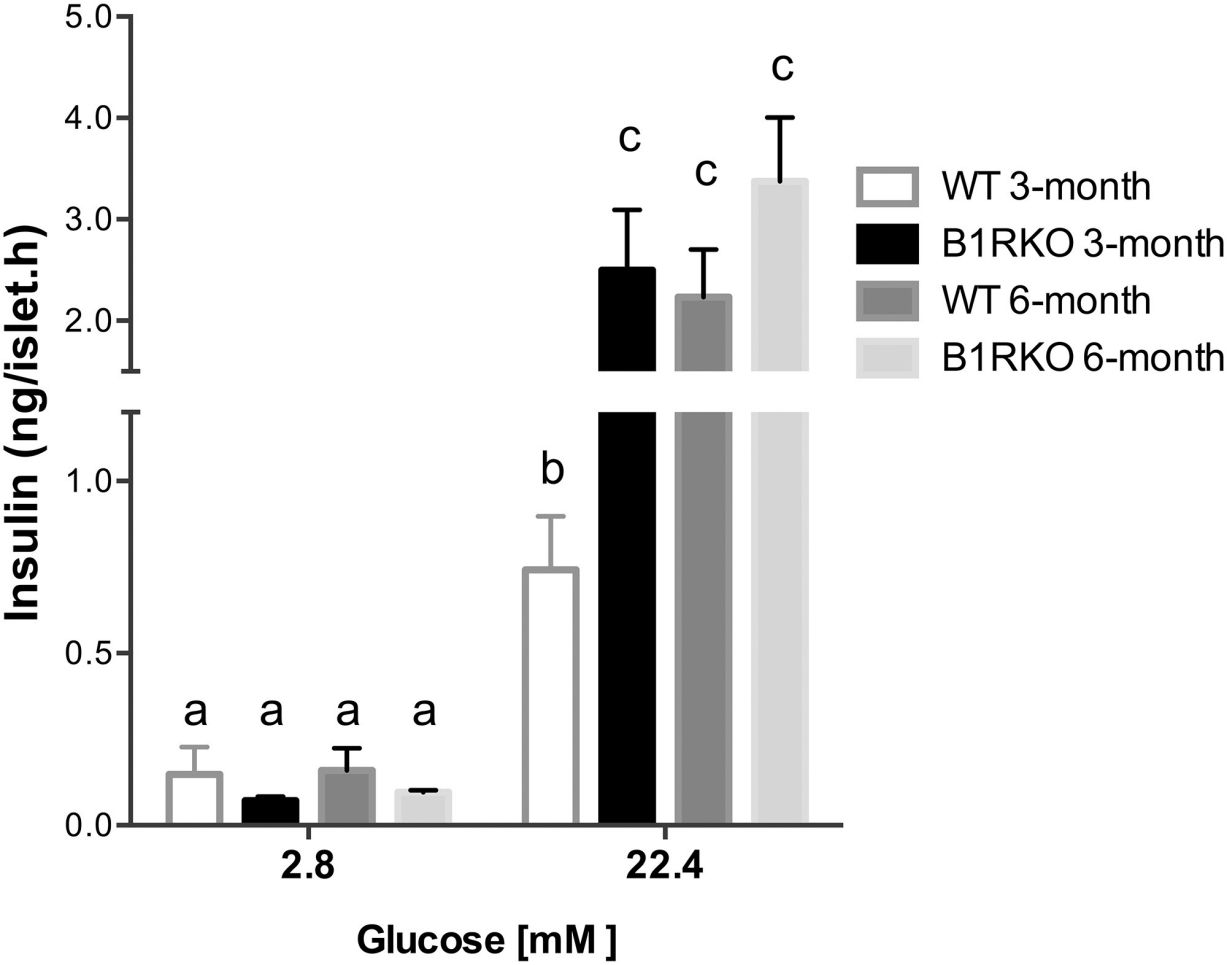
Insulin secretion in isolated islets. B1RKO and WT mice aging 3- and 6-months and fed only by SD were used to pancreatic islets isolation. Triplicates of 10 pancreatic islets from the same animal formed one experimental unit, which was repeated for each concentration. We used 4 animals of each genotype and age. Islets from 3-months old B1RKO showed an increasing insulin secretion if stimulated with high glucose concentration (22.4 mM) compared to the WT controls^b^. No difference was observed in low glucose concentration (2.8 mM) nor in older mice in the high glucose concentration. P-value tested by two-way ANOVA followed by Bonferroni post-hoc test. Different letters indicate differences in post-hoc test. WT, wild-type mice; B1RKO, B1R knockout mice; SD, standard diet; n = 4.

In order to understand the mechanisms underlying the best glucose response in B1RKO mice fed by CAF, despite the higher relative weight gain, we analyzed if such finding resulted from changes in the insulin sensitivity and/or β-cell function. First, mice were subjected to an ITT in the last week of the dietary intervention (Fig 2E). After the insulin injection, glucose decreased from 5 to 20 min., and it was lower in B1RKO vs. WT mice (Fig 2F), suggesting a reduction in the global insulin effect in knockout mice, despite their protection against glucose excursion identified in the GTT in both diets. Secondly, in order to understand whether this protection was related to a better capacity of those mice to overcome peripheral insulin resistance as a result of the highest β-cell function, the fasting blood glucose and serum insulin were assessed in order to estimate the insulin resistance (HOMA-IR) and the β-cell function (HOMA-β) (Fig 2G and 2H). Although mice fed by CAF had a higher HOMA-IR, differences between genotypes or interactions were not confirmed. On the other hand, B1RKO-CAF mice had an increased HOMA-β compared to WT mice on the same diet, with a significant genotype-by-diet interaction (Fig 2H). In order to understand whether the protection against the glucose excursion identified in the GTT in B1RKO mice was a result of the ability those animals have to overcome the increased insulin resistance with the higher insulin secretion capacity of the pancreas, it was calculated the disposition index, a surrogate estimate of β-cell function adjusted for the background insulin sensitivity. The disposition index was also higher in B1RKO-CAF animals compared to WT-CAF animals; once again, a genotype-by-diet interaction was observed (Fig 2I). To confirm such hypothesis, we cultivated pancreatic islets collected from 3- and 6-month-old mice fed a standard diet and analyzed the insulin secretion capacity in 2 different glucose concentration in culture medium. Pancreatic islets from 3-month-old BR1KO mice secreted almost 3 times more insulin when stimulated by high glucose concentration compared to WT with the same age. This difference is reduced in islets from 6-month-old mice due to the increase in insulin secretion of WT pancreatic islets at this age (Fig 3).

### Fatty infiltration in the liver during CAF

**Fig 4:**
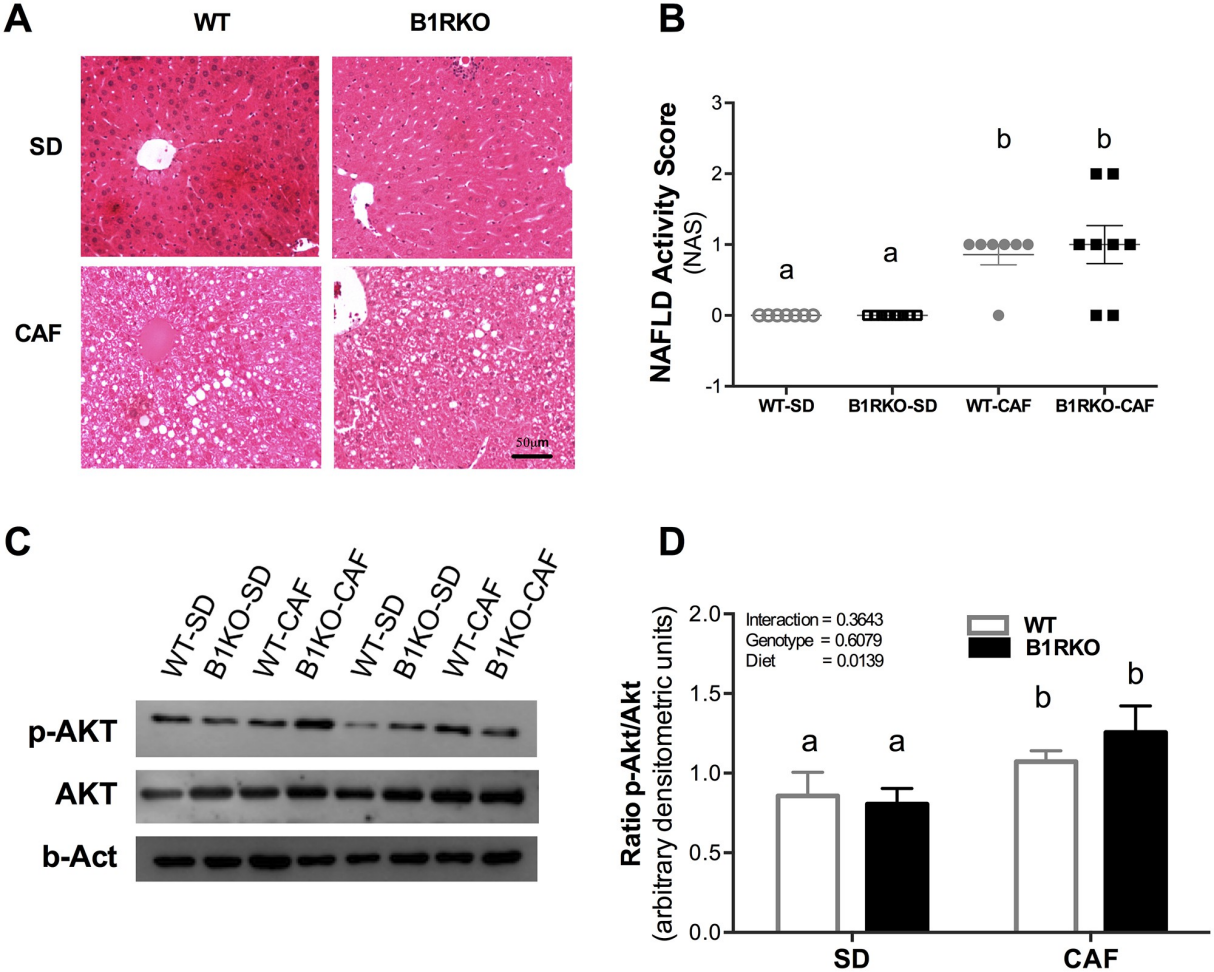
Liver fat infiltration and AKT phosphorylation according to the diet and genotype. **A)** Representative image comparing hematoxylin and eosin-stained liver sections of B1RKO and WT mice feed a CAF or standard (SD) diet (100x). **B)** NAFLD score. **C)** Representative image of p-AKT/AKT on Western blot analysis and p-AKT/AKT ratio in the liver. **D)** Analysis of p-AKT/AKT ratio in the liver. P-value tested by two-way ANOVA followed by the Bonferroni post-hoc test. Different letters indicate differences in the post-hoc test. WT, wild-type; B1RKO, B1R knockout mice; SD, standard diet; CAF, cafeteria diet, WT-SD (n = 7), B1RKO-SD (n = 8), WT-CAF (n = 7), and B1RKO-CAF (n = 10).

In order to understand how kinin B1 receptor influences fatty infiltration of the liver during the induction of weight gain with CAF, we analyzed the NAS. CAF diet induced a higher NAS score than the standard diet (Fig 4A). This diet induced a more important steatosis of hepatocytes than did the SD diet. No mice in either diet group reached a ≥ 5 score, considered significant for NASH (Fig 4B). No differences were found between genotypes, and no genotype-by-diet interaction was observed. Additionally, AKT phosphorylation in the liver was determined through Western blotting. CAF induced more AKT phosphorylation in the liver compared to the SD (Fig 4C). Such difference was not affected by genotype, and the genotype-by-diet interaction was not significant.

### Expression of glycolytic and gluconeogenic regulatory enzymes differed between diets, but not between genotypes

**Fig 5:**
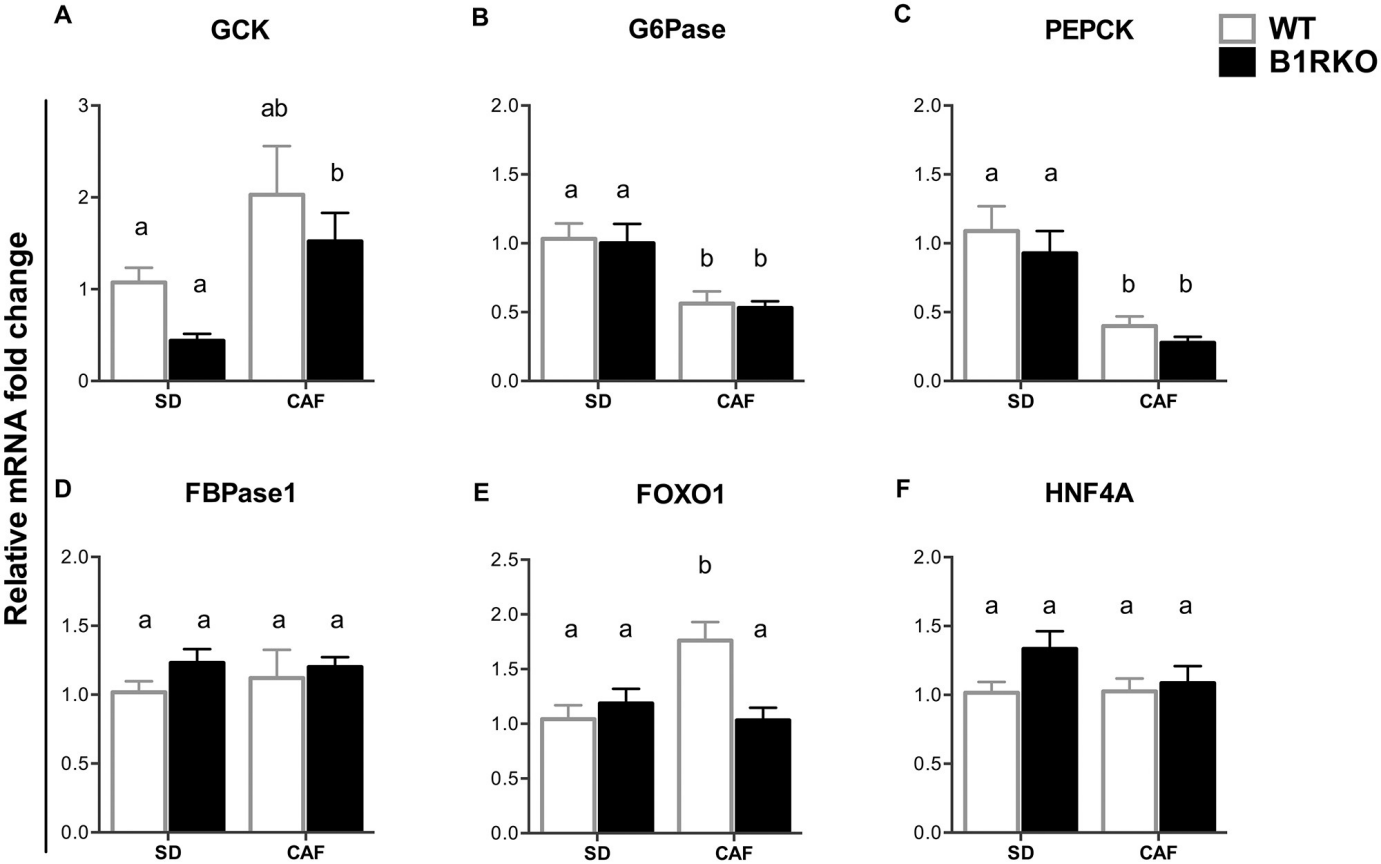
Hepatic expression of glycolysis and gluconeogenesis regulators. **A)** Glucokinase (GCK). **B)** Glucose-6-phosphatase (G6Pase). **C)** Phosphoenolpyruvate carboxykinase (PEPCK). **D)** Fructose-1,6-bisphosphatase 1 (FbP1). **E)** Forkhead box protein O1 (FOXO1). **F)** Hepatocyte nuclear factor 4 alpha (HNF4A). p < 0.05; comparing genotype (#) and diet (*); p-value by two-way ANOVA. P-value tested by two-way ANOVA followed by the Bonferroni post-hoc test. Different letters indicate differences in the post-hoc test. Data are expressed as mean ± SEM. WT, wild type; B1RKO, B1R knockout mice; SD, standard diet; CAF, cafeteria diet, n = 6.

Reduced endogenous glucose production could be another mechanism to justify the reduced glucose excursion of B1RKO mice during GTT. In order to verify this hypothesis, we studied how gene expression of regulatory enzymes involved in glucose metabolism in the liver was modulated by the dietary intervention, according to the genotype. CAF induced an increasing hepatic glucokinase (GCK) mRNA expression (Fig 5A), suggesting activation of the glycolytic pathway; simultaneously, the glucose-6-phosphatase (G6Pase) and phosphoenolpyruvate carboxykinase (PEPCK) expression was reduced in livers of mice fed with CAF, suggesting inhibition of gluconeogenesis (Fig 5B and 5C). Fructose-1,6-bisphosphatase 1 mRNA expression did not differ between groups (Fig 5D). The expression of those enzymes was not affected by the genotypes. The mRNA expression of forkhead box protein O1 (FOXO1) was decreased in the liver of B1RKO compared to WT mice fed the CAF (Fig 5E). No significant differences were observed in hepatocyte nuclear factor 4 alpha (HNF4A) expression (Fig 5F).

### CAF effect on the expression of B2 kinin receptor mRNA in the liver

**Fig 6:**
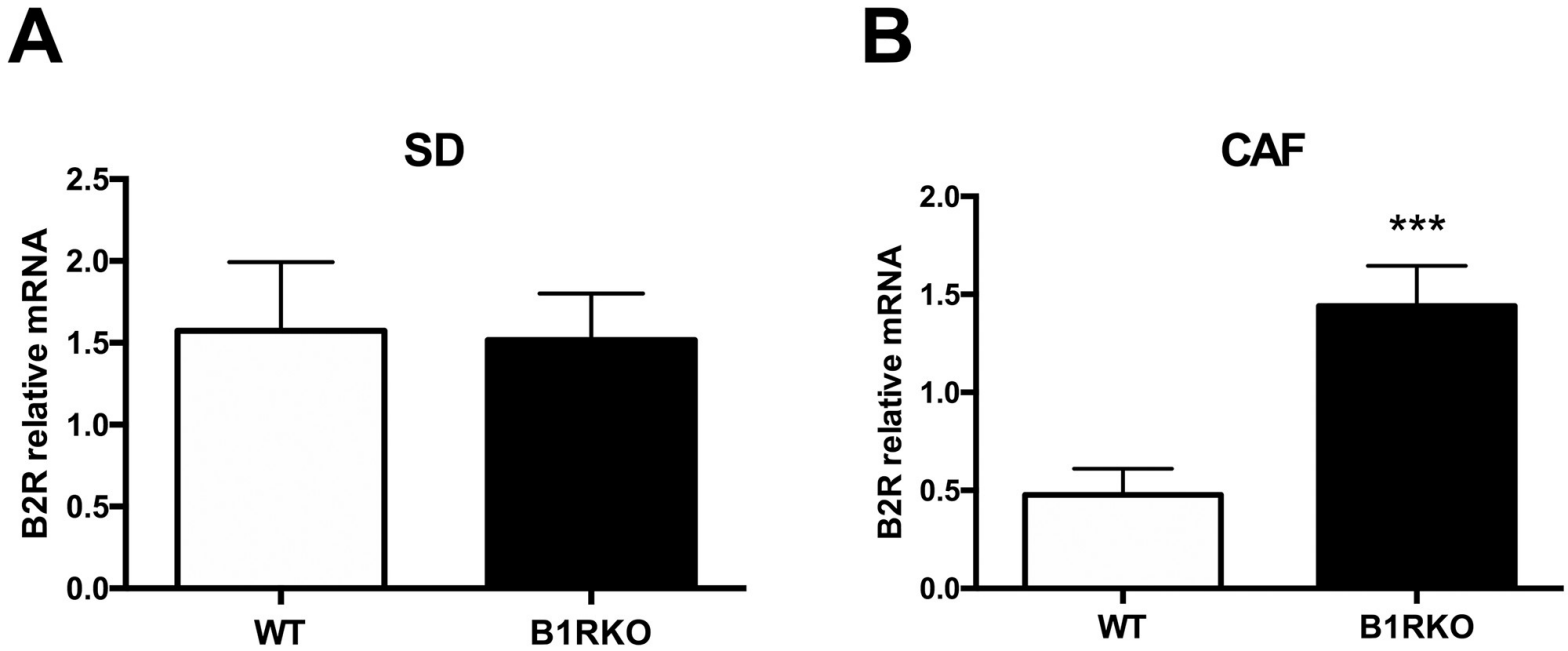
Hepatic expression of the B2 receptor. **A)** No difference was observed in mice fed by SD. **B)** B1RKO expressed more B2 receptor than in the WT controls fed by CAF. ***, p < 0.001 comparing genotype; P-value tested by two-way ANOVA followed by Bonferroni post-hoc test. Data are expressed as mean ± SEM. WT, wild type; B1RKO, B1R knockout mice; SD, standard diet; CAF, cafeteria diet, n = 6 in each group.

The compensatory increase in the B2 kinin receptor expression in the absence of B1 receptor is one possible mechanism involved in generating the difference in the glucose homeostasis between genotypes observed in the present study. We used the hepatic cDNA to check the mRNA expression. Although no difference was observed under SD, under CAF B1RKO the animals presented 3 times more expression of the B2 receptor mRNA (Fig 6).

## Discussion

The kinin B1 receptor (B1R) plays an important role in the inflammatory process and has been considered to have a role in the glucose homeostasis [15, 32–34]. In the present study, B1R knockout mice (B1RKO) were fed by the cafeteria diet (CAF), a hyperpalatable diet, rich in trans fats and refined sugar that has been used to mimic the human Western diet in rodents. While B1R deficiency did not prevent obesity, those mice were able to overcome the loss of the insulin sensitivity with a substantial increase in the insulin secretion, which can possibly explain why they had a lower glycemic response during GTT.

In order to understand the mechanisms underlying those findings, an ITT was performed after the diet intervention. CAF was able to induce insulin resistance. Differently from what would be expected based on the lower glycemic excursion of B1RKO mice during the GTT, those mice presented a reduced insulin sensitivity (K_ITT_) compared to their WT counterparts during ITT regardless the diet. Together, those findings suggest that B1RKO mice were able to increase β-cell function, and consequently, the insulin secretion to counteract their reduced insulin sensitivity. This hypothesis was suggested by assessing the disposition index, a surrogate estimate of β-cell function adjusted for the prevailing insulin sensitivity [35]. B1RKO mice exposed to the CAF had a higher β-cell response than WT-CAF mice. To confirm this hypothesis, we used a new set of mice to check the insulin release capacity of isolated pancreatic islet. We decide to use 3- and 6-month-old mice, as we had published earlier differences in the glucose metabolism and islet function in a model lacking both kinin receptors and leptin [27]. Not surprisingly, we observed an increased insulin release in the islets from 3-months old B1RKO, but only a trend in 6-month-old mice. In mice lacking both B1R and B2R and leptin [27], the islets from 3-month-old knockout mice produced three times more insulin than those of the control mice when stimulated by a high glucose concentration in the medium, similar to our findings in the present study, corroborating a possible mechanism responsible for maintaining a similar glucose response during GTT between those animals and controls, despite the increased insulin resistance observed in double knockout mice. Our results suggest that part of this effect may be related to the absence of the B1 receptor, and the mechanism involved is related to a greater ability to delivery insulin from the pancreas when challenged with glucose injection or CAF.

It was also analyzed the gene expression of enzymes responsible for regulating the hepatic metabolism. The mRNA analysis showed an increase in the glucokinase and a decrease in the PEPCK and G6Pase expression in CAF compared to SD mice, mainly when compared by the 2-way ANOVA, but no difference between genotypes was found. Those results point out to an inhibition of the gluconeogenesis, and were confirmed by the slightly increased AKT phosphorylation observed in the Western blot analysis, which is also consistent with the hyperinsulinemia caused by CAF. AKT phosphorylation is a central intracellular action caused by the insulin signaling [26]. Phosphorylated AKT phosphorylates FoxO1 cause its exclusion from the nucleus. FoxO1 is the main transcription factor activating the G6Pase and the PEPCK expression [36]. Its exclusion from the nucleus causes a reduction in the expression of those enzymes and a reduction in the gluconeogenesis [37]. Considering that CAF is a diet rich in carbohydrates and energy, those results confirm its effect on the liver metabolism. On the other hand, the lack of difference in the expression of those enzymes between genotypes shows that this was not the mechanism involved in the GTT findings, reinforcing the hypothesis of a higher insulin-secreting capacity in animals lacking the kinin B1 receptor.

In the present study, CAF was effective in inducing several metabolic responses, resulting in the disruption of energy homeostasis. Animals fed this diet presented increased energy intake, a higher relative weight gain and epididymal fat accumulation, and higher insulin resistance assessed by the HOMA-IR compared to SD-fed animals, regardless the genetic background, thus corroborating the findings of other studies [17, 38, 39]. On the other hand, we had previously demonstrated that B1RKO submitted to a high fat diet were resistant to the weight gain [5, 12]. The remarkable differences between CAF and HFD are visible in their compositions, and its reflex in obese response in different genetic model [40].

The lack of B1 the kinin receptor might increase the expression of the B2 kinin receptor as a compensating mechanism [26, 41]. Additionally, bradykinin might increase the insulin sensitivity and increase the insulin release in WT mice [27]. We analyzed the B2 mRNA expression of those mice without finding differences on the B2 kinin receptor mRNA expression in the liver of SD-fed mice. The differences found in this and in previous studies may provide a contribution as to the age differences of animals between experiments. The liver collection was performed from older than 6 months mice in the present study, and most of previously available data came from studies using 2- or 3-month-old mice [27]. On the other hand, under CAF, our mice showed increased hepatic B2 kinin receptor mRNA expression. Those results open different hypothesis as to the possible mechanism to explain at least part of the results. The increased food intake showed here could be related to changes in the central hunger and satiety control. The hypothalamus together with the reward system located in the frontal cortex and connected to the hippocampus and hypothalamus may be involved in the increases in the food intake observed in B1RKO. It was previously showed an increase in the leptin sensitivity in the hypothalamus of B1RKO fed with HFD [5]. Changes in the insulin sensitivity were also shown in similar models [6]. The injection of the B1 and B2 agonists corroborated those central influences of brain kinin receptor in the food intake regulation [42, 43]. All the data require confirmation in future studies, in order to understand how the food composition can influence the eating behavior in different genetic models.

Another finding was the induction of NAS in the liver of mice under CAF compared to those receiving SD. NAS scores were slightly increased in CAF fed mice due to the high lipid accumulation. However, this finding did not differ by genotype. We demonstrated that CAF increased the AKT phosphorylation in the liver. This result is in line with hepatic fat accumulation, suggesting the conversion of glucose into fatty acids via *de novo* lipogenesis [44]. The insulin resistance signaling pathway would result in an imbalance between *de novo* lipogenesis and β-oxidation, as well as an impairment of the hepatic gluconeogenesis pathway, thereby exacerbating the fat accumulation and the resultant hepatic steatosis [45, 46]. Fonseca at al. showed NAS protection in B1RKO fed with HFD [12]. The contrast of both, the present and previous study of our group highlights the importance of realizing that the food composition and palatability can change the metabolism related to obesity models and the genetically modified models.

This study has some limitations. We were unable to isolate pancreatic islets and perform morphometric and functional studies, in order to better understand how B1RKO animals were able to mount a better glucose response, despite their highest relative weight gain and insulin resistance in the experimental mice subjected to CAF. However, we were able to show the increased insulin release in younger B1RKO animals fed by SD. This data, along with the estimate of β-cell function and the disposition index suggest that those animals were able to overcome insulin resistance by increasing their insulin secretion. Due to the associations of CAF and kinin B1 receptor is also necessary to better analyze the inflammatory response in future studies, measuring cytokines and adipokines to understand the role of inflammation pathways in the findings here described. Another limitation of this study was that only male animals were used. When the study was designed and approved, this was a frequent practice of other experiments used as reference for the methodology. Therefore, we decided to study male animals, so that the results could be compared to previously published works.

Our results suggest that mice lacking B1 kinin receptor were able to increase their insulin secretion, overcoming the induction of insulin resistance caused by a higher weight gain while on CAF, resulting in a lower increment of the glucose levels during GTT. Taken together, those results suggest that a dissociation between induction of weight gain and protection from glucose excursion in obese B1RKO mice fed a CAF. The results suggest that the B1 receptor is a modulator of insulin release in pancreas explaining part of the glucose homeostasis changes in the B1RKO mice.

## Supporting information

S1 Raw images Raw images of western blot gels. WT-CAF: from 1 to 7; B1RKO-CAF: from 9 to 16; WT-SD: from 17 to 23; B1RKO-SD: from 33 to 42. Parts of these gels were used in Fig 4 in the main text.
(PDF)

S1 File (PDF)
