# Supplementary material for: Kinin B1 receptor deficiency protects mice fed by cafeteria diet from abnormal glucose homeostasis
Source: PLoS One. 2022 May 26;17(5):e0267845. doi: 10.1371/journal.pone.0267845 (PMC9135186; doi:10.1371/journal.pone.0267845)

S1_raw_images. Raw images of western blot gels. WT-CAF: from 1 to 7; B1RKO-CAF: from 9 to 16; WT-SD: from 17 to 23; B1RKO-SD: from 33 to 42. Parts of these gels were used in fig.4 in the main text.

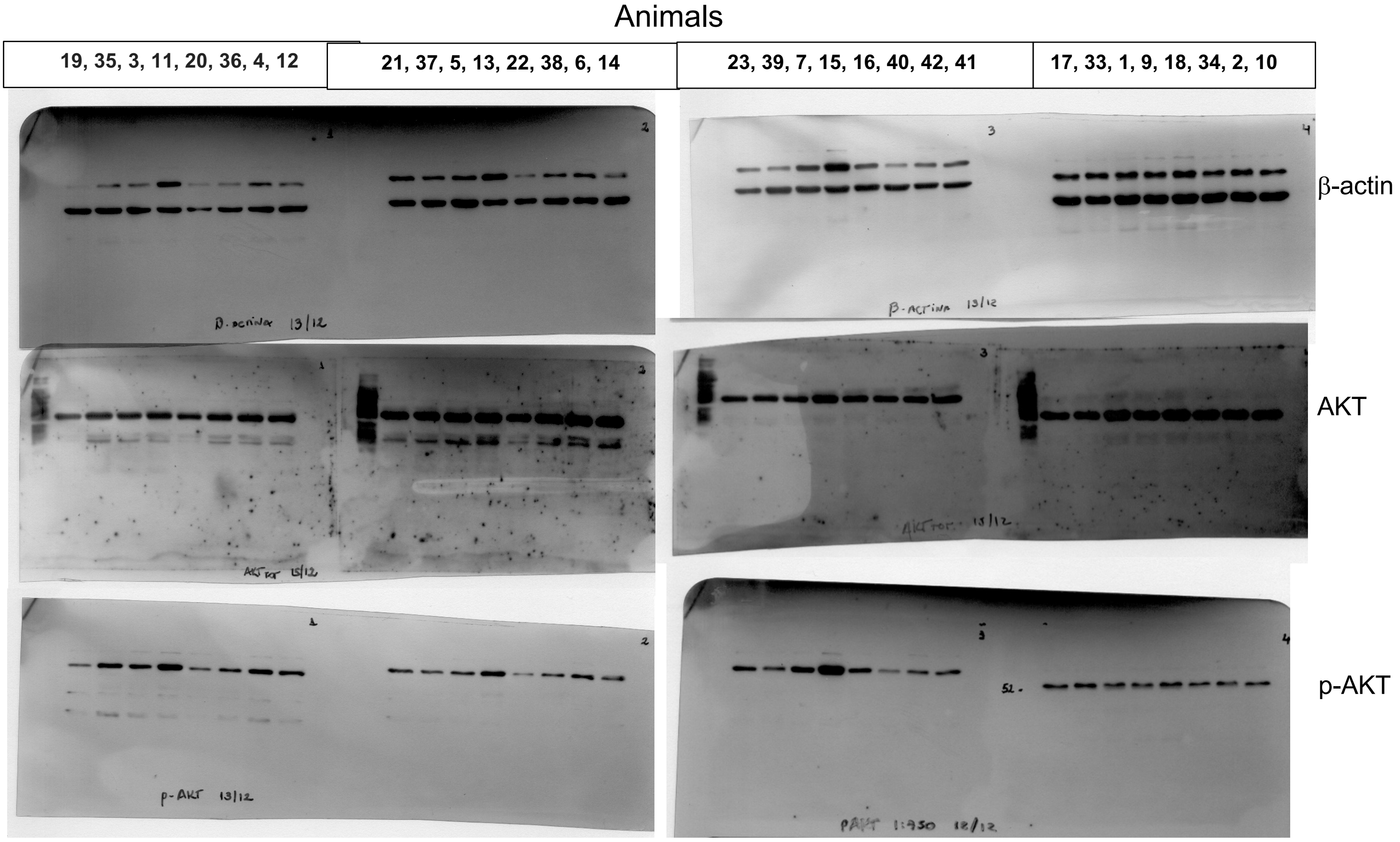

Supplement: S1 Raw images — Parts of these gels were used in Fig 4 in the main text. (PDF) [file pone.0267845.s001.pdf]
