# Supplementary figures and images for: Kinin B1 receptor deficiency protects mice fed by cafeteria diet from abnormal glucose homeostasis
Source: PLoS One. 2022 May 26;17(5):e0267845. doi: 10.1371/journal.pone.0267845 (PMC9135186; doi:10.1371/journal.pone.0267845)

# Supporting information

S2 Fig. Weekly food consumption

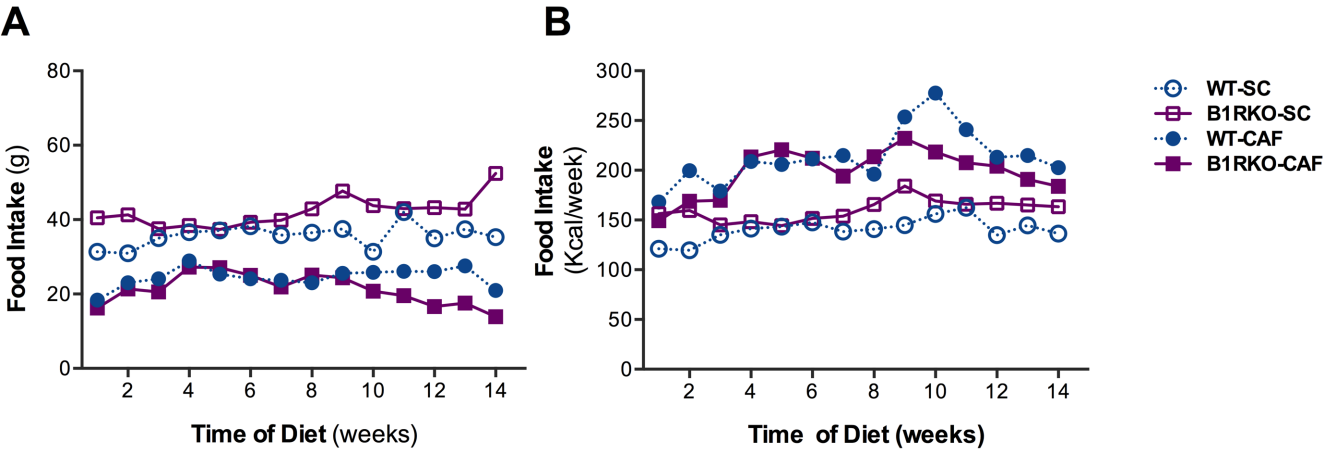

# S3 Fig. Liver AKT gel results

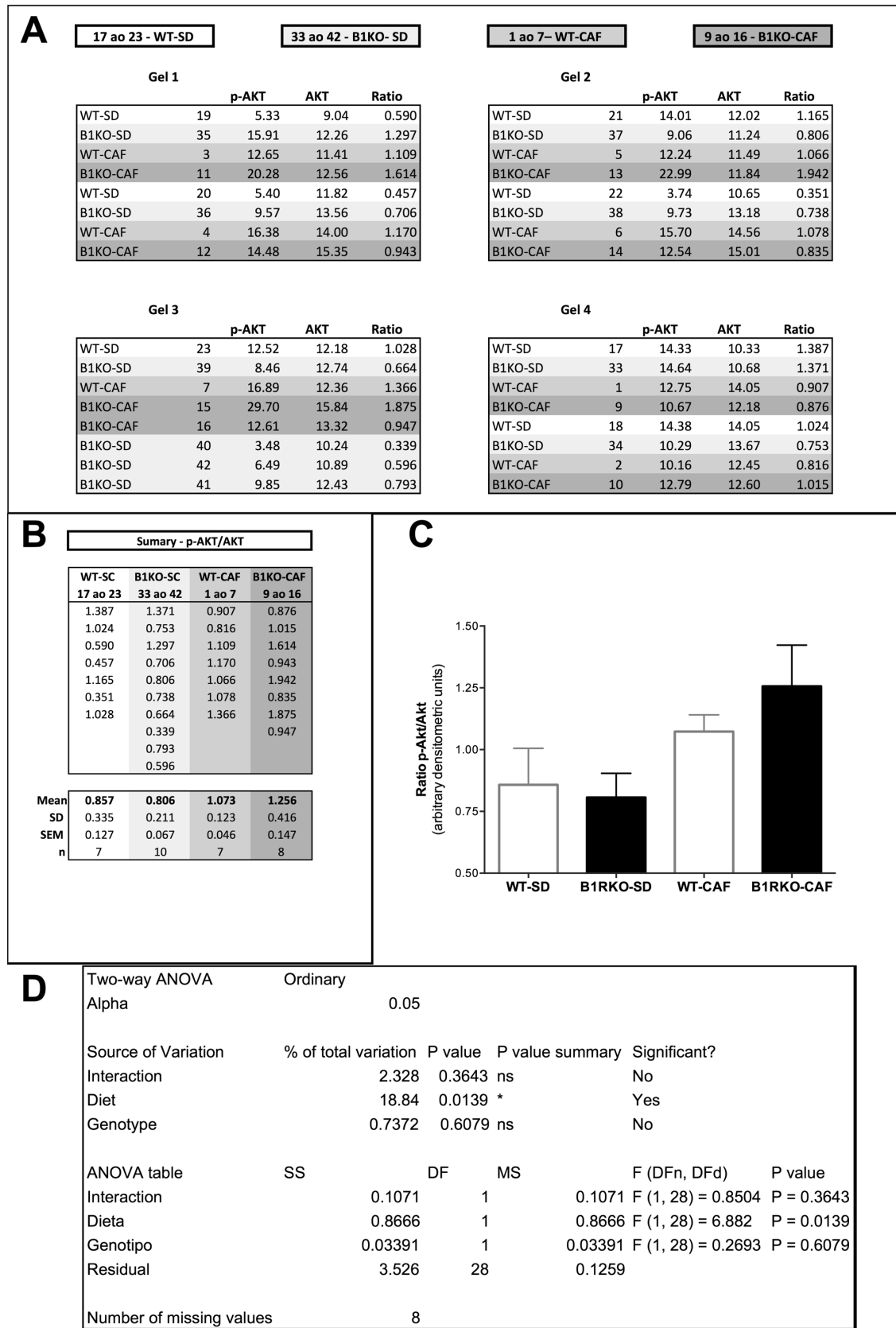

Supplement: S1 File — (PDF) [file pone.0267845.s002.pdf]
